# Correction of gene model annotations improves isoform abundance estimates: the example of ketohexokinase ( *Khk*)

**DOI:** 10.12688/f1000research.17082.2

**Published:** 2019-04-03

**Authors:** Christophe D. Chabbert, Tanja Eberhart, Ilaria Guccini, Wilhelm Krek, Werner J. Kovacs

**Affiliations:** 1 Institute of Molecular Health Sciences, ETH Zurich, Zurich, 8093, Switzerland

**Keywords:** RNA-seq, quantification, gene expression, transcriptomics, Khk, Salmon, alternative splicing

## Abstract

Next generation sequencing protocols such as RNA-seq have made the genome-wide characterization of the transcriptome a crucial part of many research projects in biology. Analyses of the resulting data provide key information on gene expression and in certain cases on exon or isoform usage. The emergence of transcript quantification software such as Salmon has enabled researchers to efficiently estimate isoform and gene expressions across the genome while tremendously reducing the necessary computational power. Although overall gene expression estimations were shown to be accurate, isoform expression quantifications appear to be a more challenging task. Low expression levels and uneven or insufficient coverage were reported as potential explanations for inconsistent estimates. Here, through the example of the ketohexokinase ( *Khk*) gene in mouse, we demonstrate that the use of an incorrect gene annotation can also result in erroneous isoform quantification results. Manual correction of the input *Khk* gene model provided a much more accurate estimation of relative *Khk* isoform expression when compared to quantitative PCR (qPCR measurements). In particular, removal of an unexpressed retained intron and a proper adjustment of the 5’ and 3’ untranslated regions both had a strong impact on the correction of erroneous estimates. Finally, we observed a better concordance in isoform quantification between datasets and sequencing strategies when relying on the newly generated *Khk* annotations. These results highlight the importance of accurate gene models and annotations for correct isoform quantification and reassert the need for orthogonal methods of estimation of isoform expression to confirm important findings.

## Introduction

Accurate measurement of mRNA expression levels is a crucial component in many modern biological studies. Common and standardized techniques such as reverse transcription real-time quantitative PCR (RT-qPCR) have remained limited in throughput, only allowing measurements for a handful of genes at a time. The emergence of Next Generation Sequencing (NGS) based protocols such as RNA-seq has overcome this limitation and enabled researchers to profile mRNA expression at the genome wide level ^1– 3^. While such experiments are now routinely performed, the subsequent bioinformatics analysis and data interpretation still pose computational challenges. As sequencing reads are currently much shorter (usually 100bp – 150bp) than most isoforms, tailored approaches are necessary to study complex events such as splicing or isoform usage switch. In addition, low number of replicates per condition together with a high dynamic range in expression levels across the genome require appropriate statistical frameworks ^4– 8^.

One common approach to analyse RNA-seq datasets consists in identifying significant changes in expression levels between two or more experimental conditions using gene-level counts ^9^. Such counts are usually obtained from the alignment of sequencing reads to a reference genome or transcriptome when available, and a subsequent counting step during which reads are assigned to annotated genes based on their mapping locations. In the absence of a large number of biological replicates, the following statistical analysis usually requires a reliable estimation of count dispersions for each gene ^4, 6– 8^. Statistical tools such as edgeR ^6^, DESeq2 ^4^ or limma ^5^ offer a panel of solutions to this problem and have been shown to perform equally well in settings where few biological replicates are available ^10^. Nevertheless, despite such progress in differential gene expression (DGE) analysis, investigating changes of splice variants and the methods for their quantification continue to be an active field of research. Being able to accurately measure such changes is all the more crucial since they are connected to various biological processes and pathologies and may be used as biomarkers and therapy targets ^11^.

Indeed, although early approaches have followed a framework similar to gene level studies and used exon level counts to implement differential exon usage analysis ^8, 12– 14^, results from such studies often remain difficult to interpret given the complexity of mammalian transcriptional units. In addition, the recent development of alignment-free transcript quantification methods has provided the possibility to efficiently and rapidly quantify each individual transcript ^15– 19^. Such approaches are indeed computationally much less demanding and faster than alignment-based methods ^15^. Moreover, they have been shown to overcome the difficulty of handling multi-mapped reads, which can create biased results in count-based analysis ^20^. Although transcript quantification estimates may be used to improve gene-level inference in DGE ^21^, testing for changes in isoform usage between conditions remains a challenging task with most approaches focusing on junction and exon read counts rather than transcript quantifications themselves. DESeq2-tximport ^21^, sleuth ^22^ and DRIMSeq ^23^ do make use of such quantifications, with sleuth incorporating estimates of inferential variances obtained during the quantification step. In contrast, DRIMSeq relies on a Dirichlet-multinomial model to estimate relative transcript usage and tests for differential transcript usage.

Regardless of the method used, several studies have now reported limitations and pitfalls associated with transcript abundance estimations ^21, 24^. Systematic errors in estimation may stem from sample-specific GC content biases ^25^, which should be accounted for when comparing conditions. In addition, a systematic assessment of quantification performance on simulated datasets also revealed a weaker accuracy in transcript abundance estimates when compared to gene abundance estimates ^21^. To explain such discrepancies, it has been suggested that certain transcript abundances cannot be reliably estimated from the data, in particular in cases where coverage is lacking in genomic regions allowing a distinction between transcripts ^21^. To our knowledge, no systematic evaluation of the impact of the presence of low coverage on such key regions has been conducted and detailed reports of such examples in real datasets are still missing.

Additionally, quantification tools rely on an input reference transcriptome to compute quantifications with the exception of Cufflinks ^17^, casper ^26^ and FlipFlop ^27^, which may be used to assemble *de-novo* transcripts. These tools are therefore limited to current gene models made available in databases such as Ensembl or RefSeq and it is unclear whether these models properly recapitulate the actual complexity of transcriptional units. An assessment of the impact of erroneous or incomplete annotations on transcript quantifications is still missing. Additionally, meticulous examination of the concordance between transcript quantification and mRNA isoforms measured using gene-tailored experimental methods has not been undertaken.

In this study, we focus on the murine ketohexokinase ( *Khk*) gene to better understand and evaluate the impact of genomic annotation on transcript quantifications. *Khk*, also known as fructokinase, is the first rate-limiting enzyme in the fructose metabolic pathway and catalyzes the conversion of fructose and ATP to fructose-1-phosphate (F1P) and ADP, respectively. Previous studies have shown that this gene predominantly expresses two usually exclusive isoforms, *KhkA* and *KhkC* that are generated via the specific excision of exon 3C and 3A, respectively ^28^ ( Figure 1A). With a greater affinity for fructose ^29^, *KhkC* is thought to be responsible for the functional role of this gene in metabolism, in particular in liver where it is highly expressed ^30^. Epidemiological and animal studies implicate overconsumption of fructose in the development of nonalcoholic fatty liver disease. While the physiological substrate of *KhkA* is unknown, several studies have highlighted the importance of *Khk* isoforms choice in the development of clear cell renal cell carcinoma (ccRCC), hepatocellular carcinoma (HCC) ^31^, and pathological cardiac hypertrophy ^32^. Given the clear importance of *Khk* isoforms expression in several disease settings, it is therefore crucial to accurately quantify these variants in order to understand relevant biological mechanisms.

**Figure 1.**
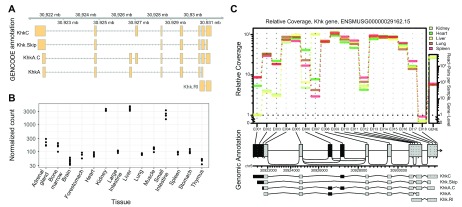
Murine *Khk* expression and splicing patterns are tissue dependent. **A** – *Khk* gene model provided by the Ensembl and GENCODE. Genomic coordinates are indicated on the top ribbon. Usual isoform names are indicated. Murine *Khk* is thought to express 4 main protein coding isoforms ( *KhkA*, *KhkC* and ENSMUST00000201571.3 and ENSMUST00000031053.14 termed *Khk.Skip* and *KhkA.C* for this study) and one isoform with a retained intron ( *Khk.RI*). **B** – Normalized *Khk* expression levels across mouse tissues (data from Li *et al.*, 2017 ^33^) using gene count tables as input. The liver, kidney and small intestine are clearly expressing *Khk* mRNA at higher levels compared with other tissues. **C** – Relative exon coverage of the *Khk* gene. Normalized exon counts are indicated in the central panel, while normalized gene expression counts are plotted to the right. Exons coloured in black were called as differentially used across all considered tissues (adjusted p value < 0.001). *KhkC* and *KhkA* expression are mutually exclusive in each tissue, with *KhkC* strongly expressed in liver and kidney while *KhkA* is expressed in heart, lung, and spleen.

By re-processing publicly available RNA-seq data ^33, 34^, we confirm that *Khk* isoforms are differentially expressed in various mouse tissues. Using DRIMSeq proportion estimations, we show that quantification of these isoforms as output by Salmon is biased by the presence of an annotated retained intron that is expressed at very low levels. We also highlight the importance of correct 3’ and 5’ UTR annotation to improve transcript quantification estimates and validate our computational findings by RT-qPCR. Finally, through the comparison of various datasets, we illustrate the importance of using correct annotations to avoid the emergence of discrepancies between library preparation protocols and datasets.

## Results

### *Khk* isoforms expression is tissue-specific

In order to assess tissue-specific expression patterns of *Khk* in mouse, we downloaded RNA-seq data generated from 14 different mouse tissues ^33^. The availability of 4 biological replicates (2 males and 2 females) per tissue enabled us to conduct a differential gene expression analysis using standard, gene-count based analytical workflows. As previously described in gene specific studies ^35^, we identified a strong tissue specific expression of *Khk* (adjusted p-value of 0 from DESeq2), with significantly higher expression levels in the liver, small intestine and kidney when compared to other tissues ( Figure 1B Underlying data: Table 1). Interestingly, gender did not impact the overall *Khk* expression levels. These results were all confirmed using the gene level estimates based on Salmon quantifications ( Extended Data Figure 1A and B). In particular, patterns of *Khk* expression across tissues were highly concordant between count-based and Salmon estimates. We also sought to evaluate changes in relative exon usage for this gene using JunctionSeq ^14^, including both exon and junction counts in our analysis. Therefore, we selected five tissues (liver, spleen, lung, heart, kidney) with known variations in *Khk* isoform usage ^30– 32, 35^ and different levels of overall expression. We clearly identified a preferential inclusion of exon 3 C (Ensembl ID ENSMUSE00000186455) in liver and kidney, as previously described, while exon 3A (Ensembl ID ENSMUSE00001361691) is preferentially retained in heart, spleen and lung (p value < 0.001) ( Figure 1C). This trend was also reflected in junctions spanning these exons ( Underlying data: Table 2). Variations in 5’ UTR were also observed, most likely resulting from alternative transcription start site usage in liver and kidney. In summary, using traditional count-based methods, we confirmed the tissue-specificity of *Khk* expression and identified exons preferentially retained in some tissues.

### An annotated, unexpressed retained intron biases *Khk* isoform quantification

As count-based methods might not always reflect the full complexity of splicing patterns and isoform diversity, we sought to quantify relative proportions of *Khk* isoforms in each tissue to fully capture the complexity of its expression patterns. The GENCODE annotation together with the Salmon ^15^ quantifier were used to obtain these estimations ( Underlying data: Table 3). Interestingly, the quantification results showed that the annotated retained intron ( *Khk.RI*) accounts for more than 15% of expressed isoforms in 8 tissues ( Figure 2A). This trend was consistently observed in DRIMSeq ^23^ proportion estimates ( Figure 2A; Underlying data: Table 3) and the raw TPM (transcripts per million) values output by Salmon ( Extended data: Figure 2). Nevertheless, examination of the coverage tracks generated from the alignment of the reads to the reference genome showed hardly any detectable expression of the transcript ( Figure 2B; Extended data: Figure 3). This observation was also supported by the differential exon usage analysis which clearly revealed very low expression levels for the only *Khk.RI*-specific exonic region, E012 ( Figure 1C; Extended data: Figure 4A). To experimentally confirm the absence of *Khk.RI* expression, we designed PCR primers amplifying fragments specific to this transcript (see Extended data: Figure 4B; Extended data: Table 1 for a list of primers used in the study) and used RT-qPCR and semiquantitative RT-PCR to measure its expression levels. *Khk*. *RI* could hardly be detected using this sensitive method ( Extended data: Figure 4C and 4D) and comparison with the expression levels in a *Khk-A/C* ^-/-^ mouse model showed that it is hardly expressed in heart, kidney, liver, lung and spleen, whereas a product could be amplified using genomic DNA as template ( Figure 2C). This experimental validation further demonstrates that the contribution of *Khk*. *RI* to the overall *Khk* expression level was overestimated during the quantification step. Taken together, these results suggest that the presence of non-expressed transcripts in the “raw” gene annotation may result in erroneous detection by a transcript quantification software.

**Figure 2.**
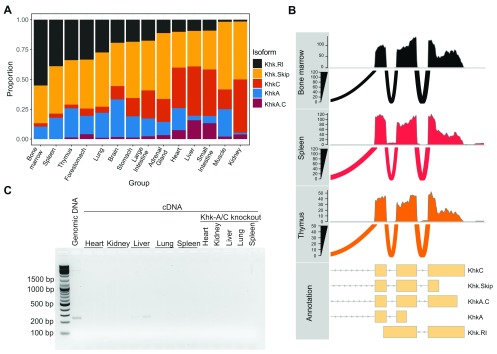
*Khk*. *RI* expression levels are overestimated using Salmon quantifications. **A** – Estimated relative *Khk* isoform expression across all tissues in mouse. Proportions were output by DRIMseq using Salmon quantification as an input. **B** – RNA-seq coverage tracks and sashimi plots highlighting the absence of *Khk*. *RI* expression in thymus, spleen and bone marrow samples. Data obtained from all biological replicates were merged prior to plotting. **C** – Products derived from semiquantitative RT-PCR analysis on cDNAs prepared from total RNA of different mouse organs using the primers specific for *Khk.RI*. Genomic DNA isolated from the liver was used as positive and RNA isolated from organs of *Khk-A/C* knockout mice as negative control. (n = 5 and products from two representative mice are shown).

### Manual update of the *Khk* transcript annotations improved quantification results

Since the current genomic annotation did not reflect *Khk* isoform expression and introduced biases in quantifications, we manually removed the *Khk*. *RI* transcript (Ensembl ID ENSMUST00000200978.1) prior to the quantification step. Despite this adjustment, inspection of the junction reads, coverage tracks, and normalised exon and junction counts derived from QoRTs ( Figure 1C) revealed discrepancies between quantification estimates and results derived from alignment-based methods ( Figure 3A and 3B; Extended Data: Figure 5). These quantification estimates also did not reflect the observations made by previous reports focusing on the characterisation of *Khk* expression patterns ^35^. An example of such discrepancy may be found examining the heart coverage tracks: while it is quite clear that the *KhkC*-specific exon 3C (Ensembl ID ENSMUSE00000186455) is hardly captured in comparison with exon 3A (Ensembl ID ENSMUSE00001361691), quantification estimates yielded a score of 39.9% and 16.7% for *KhkC* and *KhkA*, respectively ( Underlying data: Table 3). Similarly, *KhkC* estimates were inflated in lung and spleen (14.9% and 18.6% to be compared with the absence of coverage of exon 3C), as were *Khk.Skip* estimates (32%) in liver ( Underlying data: Table 3). Since such quantifications are relying on the reference transcripts provided during the indexing step ^15^, we reasoned that incorrect transcript models might be the cause of the observed discrepancies and therefore compared coverage tracks, normalised exon counts and isoform models. We identified annotated differences in 3’ end annotations between all isoforms which were not reflected on our coverage tracks ( Figure 1C and Figure 3B). As *Khk* isoforms can be identified unambiguously based on the exclusion patterns of the exons 3A and 3C and regardless of differences in UTRs, we could investigate the impact of these UTR variations on transcript quantifications. We therefore manually updated *Khk* isoform annotations to provide an identical 3’ end to all isoforms ( Underlying data: File 1 and 2) and re-estimated isoform proportions ( Figure 3A, second panel). Despite this adjustment, inspection of the proportion estimates still revealed erroneous estimations of isoform expression in particular in the case of liver where *KhkA* was detected in levels similar to *KhkC*. Further examination of the results revealed that, while some differences in 5’ end coverage in the dataset were concordant with the current gene annotation, they were not always reflected in the gene model ( Figure 1C and Figure 3A, heart, lung and spleen 5’ UTR coverage). Following a similar approach to the one described earlier for 3’ UTRs, we finally manually modified *Khk* isoforms to provide an identical 5’ and 3’ end to all listed isoforms ( Underlying data: File 3). Transcript quantification performed using this updated annotation yielded more concordant results when compared to coverage tracks and in light of previous reports, in particular for tissues such as liver ^30^, small intestine ^36^, heart ^32^, spleen and lung ( Figure 3A and 3B; Extended data: Figure 5). Interestingly, a substantial fraction of isoforms detected in kidney were still attributed to the *Khk.Skip* isoform while both junction count analysis and single gene studies reported a prevalence of *KhkC* ^35^. Additionally, we computed the relative *Khk* isoform usage using a new annotation with identical 5’ and 3’ end for all isoforms except *Khk.RI* which was retained as such in the gene model ( Figure 3A). This modification was not sufficient to remove *Khk.RI* estimation biases, with the retained intron predicted to erroneously account for 20% of the overall gene expression in bone marrow or spleen. We therefore confirmed the impact and importance of both *Khk.RI* and UTRs annotations on *Khk* isoform expression estimates.

**Figure 3.**
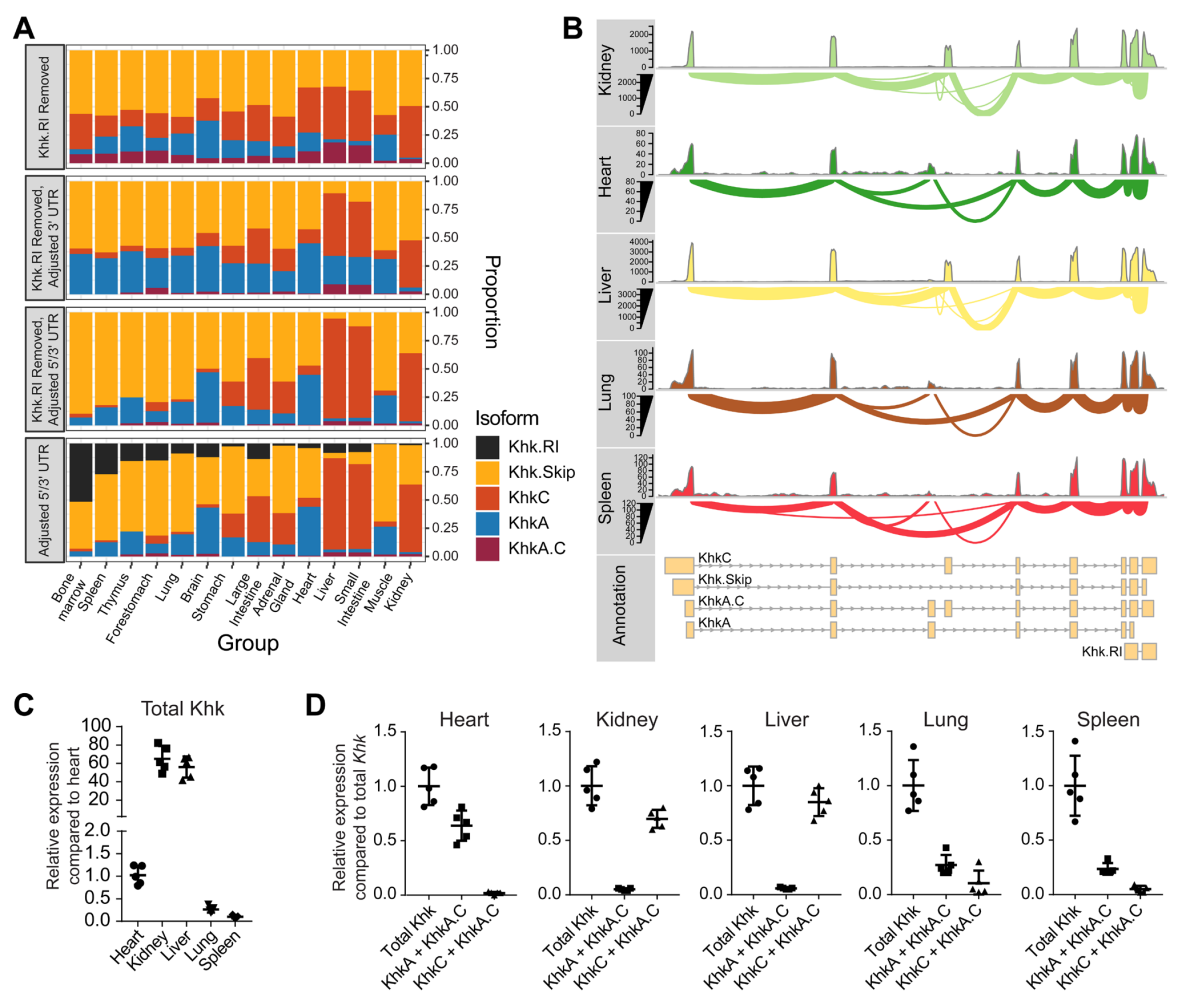
*Khk* isoform quantification estimates are improved after manual adjustment of the genomic annotation. **A** - Estimated relative *Khk* isoform expression across all tissues in mouse, using four different genomic annotations: a raw annotation after removal of the *Khk*. *RI* transcript, an annotation without *Khk.RI* and with adjusted 3’ UTR, an annotation without *Khk*. *RI* and adjusted 3’ and 5’ UTR, and an annotation with adjusted 3’ and 5’ UTR for all transcripts except *Khk.RI*. The choice of annotation greatly impacts the quantification results. **B** – RNA-seq coverage track and sashimi plots illustrating the predominance of different isoforms in spleen, lung, liver, heart, and kidney. **C** – RT-qPCR analysis of total *Khk* expression in different mouse organs. Values are expressed as fold-change compared to the expression levels obtained for the heart, which was arbitrarily defined as 1. *β-actin* was used as the invariant reference gene. Data are mean ± SD (n = 5). **D** - RT-qPCR analysis of total *Khk*, *KhkA* + *Khk.A.C*, and *KhkC* + *KhkA.C* expression in different mouse organs. Values are expressed as fold-change compared to the expression levels obtained for total *Khk*, which was arbitrarily defined as 1. Data are mean ± SD (n = 5).

To confirm the biological relevance of our newly estimated proportions, we designed primer pairs to specifically target *Khk* isoforms (see Extended data: Figure 6A and 6B; Extended data: Table 1) and evaluated their relative expression using RT-qPCR. The expression of total *Khk* was ~60-fold higher in liver and kidney compared to heart, lung, and spleen ( Figure 3C), confirming previously described findings ^35^. While we did not manage to achieve a reliable quantitative evaluation of *Khk.Skip* and *KhkA.C* expression alone, we accurately measured the expression of ( *KhkC* + *KhkA.C*) and ( *KhkA* + *KhkA.C*) in five mouse tissues and normalized it to the total *Khk* expression ( Figure 3C and 3D). We thereby confirmed a strong prevalence of ( *KhkA* + *KhkA.C*) expression in heart while ( *Khk*C + *KhkA.C*) accounted for most of the expression measured in kidney and liver ( Figure 3D). Therefore, we concluded that *KhkA.C* levels of expression were much lower than *KhkA* and *KhkC* in these three tissues and that the proportion estimates derived from our adjusted genomic annotation reflected the RT-qPCR measurements. The isoform measurements in lung and spleen highlighted low levels of *KhkA*, *KhkC* and *KhkA.C* compared to total *Khk* expression, strongly suggesting the prevalence of *Khk.Skip* expression, as observed on coverage tracks ( Figure 3B) and in the proportion estimates derived from the updated genomic annotation ( Figure 3A).

Finally, we evaluated whether the changes brought to the *Khk* transcript annotations affected the estimations of overall *Khk* expression levels. Gene level estimates obtained using quantifications based on 3 of the modified annotations were strongly correlated (Pearson, r > 0.99) with estimates derived from the raw GENCODE annotation ( Extended data: Figure 7A). In addition, the high tissue specificity of *Khk* expression was observed in all cases, with identical expression patterns between annotations ( Figure 1A, Extended data: Figure 7B).

Altogether, these findings demonstrate that UTRs and more generally 5’ and 3’ end annotation may greatly influence transcript quantification results. The resulting biases lead to the identification of isoforms that can hardly be detected in biological samples, therefore highlighting the importance of inspecting results for any given gene of interest.

### Erroneous genomic annotation of *Khk* increases discrepancies between sequencing strategies and datasets

We next investigated whether such discrepancies could be observed when using different sequencing library strategies. To do so, we artificially created a single-end dataset by removing one read for each tissue sample. We also created a short-read dataset by trimming the remaining reads to only retain the first 50bp. In both cases, we aligned reads to the reference genome and independently quantified transcript expressions using Salmon as previously described. Regardless of the sequencing strategy considered, we observed a similar overestimation of the *Khk.RI* fraction as well as discrepancies between proportion estimates and junction counts ( Extended data: Figure 8A and 8B). Both differences were corrected using the aforementioned manually curated annotations. We then compared proportion estimates between datasets for each annotation. Quite strikingly, we noted a much better agreement in transcript estimates using our manually modified annotation ( Figure 4A–D). While the use of the “raw” annotation only resulted in a 0.56 correlation (Pearson) between estimates from the paired-end and 50bp single-end libraries, the use of an updated annotation resulted in a 0.97 correlation between both platforms. This trend was also observed between paired-end and single-end and between single-end and 50bp single-end respectively ( Extended data: Figure 9A-F). To further evaluate the impact of annotations on estimate concordance across datasets, we downloaded RNA-seq data from another study profiling mRNA expression across 13 tissues ^34^. We quantified isoform usage for each tissue and compared those estimates with the ones from the 50bp single-end dataset from Li *et al.* 2017 ^33^ in order to avoid biases due to differences in read length. In total, 8 tissues could be compared between both studies. Quite strikingly, the agreement between both datasets was not high (Pearson correlation 0.72) when considering fractions derived using the original annotation ( Figure 4E). While the removal of *Khk.RI* without an adjustment of the UTR did further hinder reproducibility between both datasets ( Figure 4F), we observed a much stronger consistency of proportion estimates after UTR adjustment ( Figure 4H, Pearson correlation 0.73). However, the highest consistency between datasets was reached when using the fully updated annotation ( Figure 4G, Pearson correlation 0.91). These results therefore further underscore the importance of appropriate annotations during transcript isoform quantifications. The use of erroneous gene models may further increase discrepancies between sequencing libraries and datasets as exemplified in the case of the *Khk* gene.

**Figure 4.**
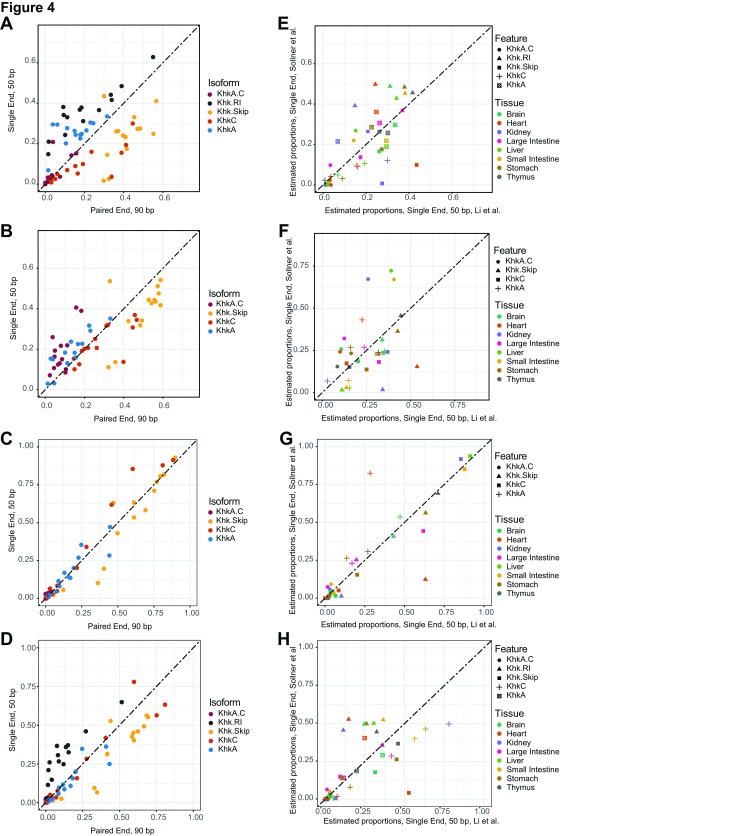
Manual adjustment of the *Khk* gene model improves the concordance between estimates across sequencing library strategies and datasets. **A** – Comparison of relative *Khk* isoforms expression estimates between the full length paired-end dataset from Li *et al.* 2017 ^33^ and the short read, single-end dataset. The estimates were generated using the naive Ensembl annotation. **B** – Comparison of relative *Khk* isoforms expression estimates between the full length paired-end dataset from Li *et al.*, 2017 ^33^ and the short read, single-end dataset. The estimates were generated using the modified Ensembl annotation where the *Khk.RI* transcript has been removed. **C** – Comparison of relative *Khk* isoforms expression estimates between the full length paired-end dataset from Li *et al.*, 2017 ^33^ and the short read, single-end dataset. The estimates were generated using the modified Ensembl annotation where the *Khk.RI* transcript has been removed and the 5’ and 3’ UTR of other transcripts were all adjusted. **D** - Comparison of relative *Khk* isoforms expression estimates between the full length paired-end dataset from Li *et al.*, 2017 ^33^ and the short read, single-end dataset. The estimates were generated using the modified Ensembl annotation where the 5’ and 3’ UTR of all transcripts except *Khk.RI* were all adjusted. **E** – Comparison of relative *Khk* isoforms expression estimates between the Li *et al.*, 2017 ^33^ dataset and the Söllner *et al.*, 2017 ^34^ dataset, using single-end, short (50bp) reads in each case. The estimates were generated using the naive Ensembl annotation. **F** – Comparison of relative *Khk* isoforms expression estimates between the Li *et al.*, 2017 ^33^ dataset and the Söllner *et al.* 2017 ^34^ dataset, using single-end, short (50bp) reads in each case. The estimates were generated using the modified Ensembl annotation where the *Khk.RI* transcript has been removed. **G** – Comparison of relative *Khk* isoforms expression estimates between the Li *et al.*, 2017 ^33^ dataset and the Söllner *et al.* dataset, using single-end, short (50bp) reads in each case. The estimates were generated using the modified Ensembl annotation where the *Khk*. *RI* transcript has been removed and the 5’ and 3’ UTR of other transcripts were all adjusted. **H** - Comparison of relative *Khk* isoforms expression estimates between the Li *et al.*, 2017 ^33^ dataset and the Söllner *et al.* dataset, using single-end, short (50bp) reads in each case. The estimates were generated using the modified Ensembl annotation where the 5’ and 3’ UTR of all transcripts except *Khk.RI* were all adjusted.

## Discussion

Gene and transcript quantifications are essential steps in many genomic studies where the characterization of gene expression patterns is of biological relevance. The recent development of quasi-mapping methods such as Salmon ^15^ has drastically improved the computational speed of these quantifications steps while relying on reduced computational power. However, unlike more traditional and alignment-based methods, they strongly rely on the provided genomic annotation. Through the example of *Khk* gene expression in mouse, we describe the importance of using a properly curated annotation to avoid biases and erroneous isoform proportion estimates. We show that the inclusion of an annotated, yet not detected retained intron ( *Khk.RI*) was sufficient to wrongly predict isoform usage in several tissues. We also found that differences in 5’ and 3’ end annotations may result in inaccurate transcript quantifications. Manual adjustment of such differences resulted in a better agreement between isoform proportion estimates, coverage tracks inspections, junction counts and qPCR results in at least 3 tissues. Both the removal of *Khk.RI* and UTR adjustments were necessary to reach this concordance between profiling methods. Finally, comparison of these estimates across different datasets and sequencing library strategies revealed that the use of a corrected annotation strongly improves the reproducibility of estimations between each dataset.

The use of gene or exon level counts to assess differences in gene expression or exon usage between conditions has been described as a robust method by several independent studies ^8^. Recent reports ^21^ have also emphasized the reliability of gene-level quantification estimates and their biological relevance. It was therefore reassuring to observe a very good agreement between both methods when assessing the tissue specificity of *Khk* expression in this dataset. Identification of higher levels of expression in liver, small intestine and kidneys reflects previously described findings ^35^. We thereby provide further evidence of the reliability of gene quantification, albeit at the single gene level. Additionally, the variations observed in expression levels across tissues together with the previously reported alternative splicing events make *Khk* an ideal gene to study performance of bioinformatics tools.

We identified strong discrepancies between proportion estimates and coverage tracks when quantifying *Khk* isoforms while relying on the original GENCODE/Ensembl annotation. In particular, the annotated retained intron *Khk.RI* (ENSMUST00000200978.1) was identified as a predominantly expressed isoform in several tissues while hardly any read could be mapped to the genomic region specific to this isoform. RT-qPCR validations confirmed the extremely low levels of *Khk.RI* in 5 mouse tissues. Previous work relying mostly on simulated data showed that in the case of lowly expressed genes, transcript-level estimates lack accuracy ^21^. However, through the instance of *Khk*, we provide a concrete example of a strong overestimation in transcript quantification and show that this is not limited to tissues with very low expression of the corresponding gene.

Differential exon usage analysis clearly revealed, as expected, a preferential usage of either exon 3A (Ensembl ID ENSMUSE00001361691) or 3 C (Ensembl ID ENSMUSE00000186455) in various tissues ^35^. The discovery of a potential change in 5’ transcription start site, while not previously described for *Khk*, further underscores the importance of alternative start and termination sites in transcript isoform diversification in mammals ^37^. Interestingly, inspection of the usage of other exons revealed that these new start sites are most likely specific to the retention of exon 3A or 3C, therefore suggesting that the determination of the isoform choice for *Khk* could be achieved solely by considering junction and exon counts in this region.

This idiosyncrasy was further confirmed by the various isoform proportion estimations performed using updated annotations of the *Khk* gene. Estimations reflected experimental measurements, junction counts and coverage tracks only when both 5’ and 3’ end annotations were harmonized across all annotated isoforms. Importantly, when using the naive GENCODE annotation, Salmon and DRIMseq failed to reliably quantify isoform proportions. It is also important to note that the curated RefSeq *Khk* gene model differs from GENCODE as it is missing *Khk.RI*, *Khk.Skip* and the 3’ and 5’ UTRs of all curated transcripts are identical. While this would be a close configuration to the optimal annotation presented in this study, but the absence of *Khk.Skip* in the gene model would result in erroneous quantifications as well. Such misestimations are likely to be observed in other genes for which current annotations are either limited or inaccurately reflect experimental measurements. However, systematic harmonisation of all UTRs across annotated transcripts might not be a general approach, especially in cases when such differences are reflecting tissue-specific expression patterns ^37^.

During the preparation of this manuscript, a preprint from Soneson *et al*. reported a similar observation and proposed the creation of a new index to flag such problematic genes ^38^. While the current manuscript strongly emphasizes the role of 3’ UTRs in the emergence of estimation biases, we could pinpoint at least one example where 5’ UTRs play a similar role in the issue. The use of the JCC (Junction Coverage Compatibility) score introduced by Soneson *et al*. will be greatly useful to prevent misinterpretation of transcriptomics studies in the future but will tie quantifications to the results of computationally demanding alignment methods ^38^. Improvement of current genomic annotations might ultimately offer an alternative as they will allow for the sole use of fast quantification algorithms. This might partially be achieved using transcript catalogues obtained from large scale studies such as CHESS ^39^ even though Soneson *et al* ^38^ reported very little to no improvement in their JCC scores using these new annotations.

Using an additional dataset from Söllner *et al.*, 2017 ^34^ and in-silico single-end and short single-end datasets from Li, B *et al*. 2017 ^33^, we showed that such updated annotations have the potential to reduce discrepancies between methods and experiments. While this is only exemplified at the level of the *Khk* gene, it is very likely that other instances will emerge as new metrics such as JCC will enable scientists to flag problematic genes. The main results of this study exclusively focus on the use of Salmon as a quantification software and DRIMSeq to estimate relative proportions, in particular as recent reports have suggested that most quantification pipelines might perform similarly ^24^. To complement our main findings, we estimated transcripts abundance in the Li *et al.*, 2017 dataset using StringTie ^40^, as an example of a tool relying on the construction of a splicing graph to quantify isoforms. When using the GENCODE annotation as a guide for quantification, we found that StringTie overestimated the *Khk.RI* expression in a fashion similar to Salmon. This is in concordance with the report from Soneson *et al.* ^38^. In addition, we used StringTie without any supporting annotation to enable transcript assembly from the alignments. Inspection of the resulting newly assembled transcripts showed that *Khk.RI* could not be detected ( Extended Data, Figure 10). Nevertheless, *KhkA.C* was also not identified in the dataset while junction counts clearly indicate that it could be detected in a handful of tissues, albeit with low expression levels. We therefore suggest that the issue reported here in the case of Salmon might be commonly found across quantification softwares, and it will be interesting to assess whether similar biases may arise with more tools. Results from Soneson *et al.* strongly indicate that this is the case, at least for a group of human genes ^38^.

Finally, the results presented in our study will provide a valuable resource to the scientific community investigating the role of fructose metabolism and *Khk* in mammals. As each *Khk* isoform might harbor different functions, the complete mapping of their usage across tissues will help further pinpoint their role in different biological contexts. Our analysis was conducted on mouse tissues but further exploration of the results presented in Reyes *et al.* 2017 ^37^ also showed that exons 3A and 3C of *KHK* are selectively included in human tissues, across individuals. Refining our understanding of these expression patterns in human will be critical in particular as the KHK protein product has already been identified as a promising target in the treatment of non-alcoholic steato-hepatitis (NASH) ^41^. This study provides important background information to improve the results of the transcriptomic work that might therefore be necessary in the future.

## Methods

### Mice

C57BL/6J were obtained from The Jackson Laboratory, while *KhkA/C ^-/-^* mice, which are of C57BL/6 background and are lacking both ketohexokinase-A and ketohexokinase-C, were obtained from R. Johnson (University of Colorado) and used as negative control. All mice were housed in a pathogen-free facility at the ETH Phenomics Center (EPIC) under standard conditions (12 h light and 12 h dark cycle) with free access to food and water. 3 female and 2 male C57BL/6J mice and 2 male *Khk-A/C ^-/-^* mice were euthanized with CO _2_ at the age of 6 weeks and heart, kidney, liver, lung, and spleen were subsequently removed and shock-frozen in liquid nitrogen. The mice did not suffer during the euthanasia with CO _2_; the mice were placed into a chamber that contained room air and then CO _2_ was gradually introduced with no more than 6 psi to displace at least 20% of the chamber volume per minute. All protocols for animal use and experiments were reviewed and approved by the Veterinary Office of Zurich (Switzerland).

### Evaluation of *Khk* isoform expression *in vivo*

Total RNA from C57BL/6J and *KhkA/C ^-/-^* mice was prepared from frozen tissues with RNeasy Mini Kit (QIAGEN, Hilden, Germany) and treated with DNase I to remove traces of DNA. First-strand complementary DNA (cDNA) was synthesized with random hexamer primers using the High-Capacity cDNA Reverse Transcription Kit (Cat. No. 4368813; Applied Biosystems). Quantitative reverse transcription PCR (RT-qPCR) was performed on a Roche LightCycler 480 in duplicates using 10 ng cDNA and the 2x KAPA SYBR FAST qPCR Master Mix LC480 (Sigma). Thermal cycling was carried out with a 5 min denaturation step at 95 °C, followed by 45 three-step cycles: 10 sec at 95 °C, 10 sec at 60 °C, and 10 sec at 72 °C. Finally, melt curve analysis was carried out to confirm the specific amplification of a target gene and absence of primer dimers. Relative mRNA amount was calculated using the comparative threshold cycle (C _T_) method. β *-actin* was used as the invariant reference gene. The PCR amplification efficiency of RT-qPCR primer sets was determined with serial dilutions of liver cDNAs and was similar for all primer sets. Semiquantitative RT-PCR was performed using Phusion High-Fidelity DNA Polymerase (New England Biolabs) and the following 3-step amplification protocol: 30 sec at 98 °C (denaturation), 30 cycles of 10 sec at 98 °C, 30 sec at 63 °C, and 40 sec at 72 °C, and a final elongation step for 5 min at 72 °C. PCR products were evaluated after gel-electrophoresis. Primer sequences are listed in Extended data: Table 1.

### Modification of *Khk* transcript annotation

A fasta file containing all manually modified *Khk* transcripts was created. All exonic sequences used to modify the gene model were downloaded from the Ensembl website. A fasta file containing nucleotide sequences of all transcripts from the GENCODE M14 annotation was loaded into R using the Biostrings v 2.46.0 package. All original *Khk* transcripts were removed from the DNAStringSet object and the updated transcripts were then added. The resulting annotation was written to a fasta file to generate Salmon indexes (see following sections for more details).

### RNA-seq data processing and alignment

Fastq files from Li *et al.*, 2017 ^33^ and Söllner *et al.*, 2017 ^34^ were downloaded from SRA using the sra-tools software v2.7.0 ^42^. As they only provided two replicates (instead of 4), we excluded the testis and ovary samples from Li *et al.*, 2017 ^33^. Additionally, due to very low library complexities, we also excluded the pancreatic samples from Söllner *et al.*, 2017 ^34^. Reads were aligned to the M14_GRCm38.p5 reference genome using STAR 2.4.2a ^43^ together with the GENCODE M14 annotation (STAR was run with default parameters). The search for novel junctions was allowed during the mapping step. Gene, exon and junction level read counts were generated using the QoRTs software v1.2.42 ^44^ after excluding reads with multiple alignments (MAPK score less than 255). All workflows were orchestrated using Snakemake v 3.13.3 ^45^.

### Transcript quantifications

Salmon 0.9.1 ^15^ and StringTie 1.3.3b ^40^ were used for transcript quantifications. In the case of Salmon, indexes were built from each fasta files using the default quasi-mapping mode and a k-mer of length 31 as recommended in the software documentation. Transcript isoforms were quantified using the default VEBM algorithm. Library types were inferred by the software. Sequence and GC bias corrections were performed during each quantification (--seqBias and –gcBias options) and 100 bootstraps were run to estimate the variance of abundance estimates.

In order to quantify transcripts using StringTie, alignments to the reference genome were first re-generated as described in the “RNA-seq data processing and alignment” section with the additional STAR flag “--outSAMstrandField intronMotif”. Quantifications were then performed with and without the GENCODE annotation used as a guide (-G option in StringTie). All other parameters were set to default values. Newly assembled transcripts were then merged using the transcript merge mode (--merge) with default parameters.

### Differential gene expression

Gene count tables were loaded into R (v 3.4.1) as a DESeq2 ^4^ object to conduct differential gene expression analysis. For the purpose of differential gene expression analysis, we only retained features labelled as “gene” in the GENCODE annotation and of type *protein_coding*, *antisense*, *sense_intronic*, *3prime_overlapping_ncRNA*, *sense_overlapping* or *non_coding* in order to exclude transcription products requiring specific library preparations to be accurately measured. Genes with very low counts were excluded from downstream analysis: the threshold was set at 50 mapped reads across all samples, corresponding to less than one read per sample on average. Estimated size factors were used to correct for differences in library size. Following the standard DESeq2 workflow ^46^, changes in gene expression were modelled using a variable accounting for differences in tissue of origin for each sample. A Likelihood Ratio Test (“LRT” option in DESeq2) was performed to compare this model to a reduced model consisting of only an intercept. Results were extracted with the DESeq2 *results* function and multiple testing correction was performed using the Benjamini Hochberg procedure ^47^.

### Differential exon and junction usage

Exon and junction count tables from QoRTs were loaded in R (v 3.4.1) using the JunctionSeq (v 1.8.0) package ^14^. Changes in exon usage were modelled using the tissue of origin as a main variable. Size factors and dispersions were estimated using default parameters (options “byGenes” and “advanced” respectively, see the JunctionSeq vignette for more details). Dispersion function fits, test for differential usage and estimation of effect sizes were also run using default parameters. Final test results were extracted using the *writeCompleteResults* function. Feature-level p values were adjusted using the Benjamini Hochberg procedure ^47^. Only features with an adjusted p-value below 0.05 were retained.

### Estimation of relative isoform proportions

Transcript isoform quantifications from Salmon were loaded into R (v 3.5.1) using the tximport package ^21^. Relative isoform proportions and differential transcript usage were then modelled using a Dirichlet-multinomial model as described in Nowicka *et al.*, 2016 ^23^ and implemented in the DRIMSeq package (v 1.6.0). Model precisions were estimated by the *dmPrecision* function run with default parameters, except for the search grid ranges which were set to -15 and 15. For each sample, feature proportions were then computed using the *dmFit* function (default parameters).

### Data visualization

Genomic annotations and coverage tracks were plotted using the Gviz package (v 1.22.3) ^48^. Data from all available biological replicates were pooled together prior to plotting each track. Results from differential exon and junction usage were visualized using JunctionSeq. Other plots were generated with the ggplot2 package.

## Data availability

### Underlying data

Fastq files from Li *et al.*, 2017 ^33^ are available from: https://www.ncbi.nlm.nih.gov/bioproject/?term=PRJNA375882

Fastq files from Söllner *et al.*, 2017 ^34^ are available from: https://www.ebi.ac.uk/arrayexpress/experiments/E-MTAB-6081/

Scripts used to generate the analysis presented in this paper are freely and publicly available on Github: https://github.com/chbtchris/Khk_quantifications (Archived scripts: http://doi.org/10.5281/zenodo.2583233 ^49^).

All underlying data are available at: https://doi.org/10.17605/OSF.IO/NMKFA ^50^. Data are available under the terms of the Creative Commons Zero "No rights reserved" data waiver ( CC0 1.0 Public domain dedication). Files available are as follows:

- **Table 1:** Normalized *Khk* counts for each sample (and therefore tissue) considered.
- **Table 2:** Normalized counts for each exonic regions and tissue group output by JunctionSeq.
- **Table 3:** Relative *Khk* isoform expression across all tissues using the different genomic annotations.
- **Table 4:** Relative *Khk* isoform expression across all tissues using the GENCODE annotation and StringTie as a quantification software.
- **File 1:** Fasta file containing the sequences of *Khk* transcript isoforms downloaded from GENCODE.
- **File 2:** Fasta file containing the sequences of *Khk* transcript isoforms after removal of the *Khk.RI* isoform and adjustment of the 3’ UTR for all remaining transcripts.
- **File 3:** Fasta file containing the sequences of *Khk* transcript isoforms after removal of the *Khk.RI* isoform and adjustment of the 5’ and 3’ UTR for all remaining transcripts.
- **File 4:** Fasta file containing the sequences of *Khk* transcript isoforms after adjustment of the 5’ and 3’ UTR for all remaining transcripts except *Khk.RI*.

### Extended data

All extended data are available at: https://doi.org/10.17605/OSF.IO/NMKFA ^50^. Data are available under the terms of the Creative Commons Zero "No rights reserved" data waiver ( CC0 1.0 Public domain dedication). Files available are as follows:

- **Table 1:** List of qPCR primers used in this study
- **Figure 1:** A - Normalized *Khk* expression levels across tissues in mouse (data from Li *et al.* 2017 ^33^) using transcript abundance estimates from Salmon as input. Similar to observations made using raw counts, the liver, kidney and small intestine are clearly expressing *Khk* mRNA at higher levels compared to other tissues. B – Comparison of gene expression estimates using count tables and transcript abundance estimates.
- **Figure 2:** Transcript Per Million (TPM) values for each annotated isoform of the *Khk* gene as returned by Salmon. The naive Ensembl annotation was used to estimate the abundances.
- **Figure 3:** RNA-seq coverage tracks and sashimi plots highlighting the absence of *Khk.RI* expression in all the tissues investigated in this study. Data obtained from all biological replicates were merged prior to plotting.
- **Figure 4:** A – Normalized fractions of reads mapped to all exonic region overlapping the *Khk.RI* transcript. E12, which is the only *Khk.RI* specific region, clearly show a reduced coverage when compared to other regions. Exonic regions were determined using the JunctionSeq package and their associated genomic coordinates are available in Underlying Data, Table 2. B – Schematic representing the location of *Khk.RI* specific primer targets. C – Amplification plots of RT-qPCR analysis of *Khk*. *RI* expression in different mouse organs (n = 5 mice). Note that the Ct or threshold cycle value at which the fluorescence generated within a reaction crosses the threshold, a numerical value assigned for each run reflecting a statistically significant point above the calculated baseline, is very high (> 40) and can be considered as noise. D – Melt curves from RT-qPCR analysis of *Khk.RI* expression in different mouse organs.
- **Figure 5:** RNA-seq coverage track and sashimi plots illustrating the predominance of different isoforms in each tissue inspected in this study. Data obtained from all biological replicates were merged prior to plotting.
- **Figure 6:** A – Schematic representing the location of *Khk.A, KhkC, KhkA.C* and total *Khk* specific primer targets. B – Amplification plots of RT-qPCR analysis of total *Khk*, *KhkC*, and *KhkA* expression in different mouse organs (n = 5 mice). C – Melt curves from RT-qPCR analysis of total *Khk*, *KhkC*, and *KhkA* expression in different mouse organs.
- **Figure 7:** A - Normalized *Khk* expression levels across mouse tissues (data from Li *et al.*, 2017 ^33^) using the transcript abundance estimates from Salmon using four annotations used in the study: a naive annotation after removal of the *Khk*. *RI* transcript, an annotation without *Khk.RI* and with adjusted 3’ UTR, an annotation without *Khk*. *RI* and adjusted 3’ and 5’ UTR, and an annotation with adjusted 3’ and 5 UTR for all transcripts except *Khk.RI.* B – Comparison of the normalized *Khk* expression levels obtained using four different annotations used in the study: a naive annotation after removal of the *Khk*. *RI* transcript, an annotation without *Khk.RI* and with adjusted 3’ UTR, an annotation without *Khk*. *RI* and adjusted 3’ and 5’ UTR, and an annotation with adjusted 3’ and 5’ UTR for all transcripts except *Khk.RI.* All reported coefficients are Pearson correlations.
- **Figure 8:** A - Estimated relative *Khk* isoform expression across all tissues in mouse, using the single-end dataset created from Li *et al.*, 2017 ^33^. Three different genomic annotations were considered: a naive annotation, a naive annotation after removal of the *Khk.RI* transcript, and an annotation without *Khk.RI* and with adjusted 3’ and 5’ UTR. The choice of annotation greatly impacts the quantification results. B - Estimated relative *Khk* isoform expression across all tissues in mouse, using the 50bp single-end dataset created from Li *et al.*, 2017 ^33^. Three different genomic annotations were considered: a naive annotation, a naive annotation after removal of the *Khk.RI* transcript, and an annotation without *Khk.RI* and with adjusted 3’ and 5’ UTR. The choice of annotation greatly impacts the quantification results.
- **Figure 9:** A – Comparison of relative *Khk* isoforms expression estimates between the full length paired-end dataset from Li *et al.*, 2017 ^33^ and the full length, single-end dataset. The estimates were generated using the naive Ensembl annotation. B – Comparison of relative *Khk* isoforms expression estimates between the full length paired-end dataset from Li *et al.*, 2017 ^33^ and the full length, single-end dataset. The estimates were generated using the modified Ensembl annotation where the *Khk*. *RI* transcript has been removed. C – Comparison of relative *Khk* isoforms expression estimates between the full length paired-end dataset from Li *et al.*, 2017 ^33^ and the full length, single-end dataset. The estimates were generated using the modified Ensembl annotation where the *Khk*. *RI* transcript has been removed and the 5’ and 3’ UTR of other transcripts were all adjusted. D – Comparison of relative *Khk* isoforms expression estimates between the full length single-end dataset generated from Li *et al.*, 2017 ^33^ and the short read, single-end dataset. The estimates were generated using the naive Ensembl annotation. E – Comparison of relative *Khk* isoforms expression estimates between the full length single-end dataset generated from Li *et al.*, 2017 ^33^ and the short read, single-end dataset. The estimates were generated using the modified Ensembl annotation where the *Khk*. *RI* transcript has been removed. F – Comparison of relative *Khk* isoforms expression estimates between the full length single-end dataset generated from Li *et al.*, 2017 ^33^ and the short read, single-end dataset. The estimates were generated using the modified Ensembl annotations where the *Khk*. *RI* transcript has been removed and the 5’ and 3’ UTR of other transcripts were adjusted.
- **Figure 10:** A - Estimated relative *Khk* isoform expression across all tissues in mouse using StringTie as a quantification software and the naïve GENCODE annotation. B – Comparison of the *Khk* gene model provided by GENCODE and the newly assembled transcripts from StringTie. Transcripts assembled by StringTie are colored in red (transcript id 25257.1, 25257.2, 25257.3). No transcript could be associated to *Khk.RI* or *Khk.Skip* using the StringTie approach.

## Author information

Christophe D. Chabbert is currently at Roche Innovation Center Zurich, Wagistrasse 10, 8952 Schlieren, Switzerland.
