# Supplementary material for: Correction of gene model annotations improves isoform abundance estimates: the example of ketohexokinase ( Khk)
Source: F1000Res. 2019 Apr 3;7:1956. Originally published 2018 Dec 19. [Version 2] doi: 10.12688/f1000research.17082.2 (PMC6464065; doi:10.12688/f1000research.17082.2)
Supplement: Peer review history

# Peer review: Reviewer response for version 2

**DOI:** 10.5256/f1000research.20430.r46714

**Published:** 2019-04-26

**Authors:** Stephen N Floor, Luisa Mayumi Arake de Tacca

**Affiliations:** 1 Comparative Biochemistry Program, University of California, Berkeley, Berkeley, CA, USA; 2 Department of Cell and Tissue Biology (CTB), University of California, San Francisco (UCSF), San Francisco, CA, USA

The authors have addressed review comments - thanks!

We have read this submission. We believe that we have an appropriate level of expertise to confirm that it is of an acceptable scientific standard.

# Peer review: Reviewer response for version 2

**DOI:** 10.5256/f1000research.20430.r46712

**Published:** 2019-04-12

**Authors:** Magnus Rattray

**Affiliations:** 1 Faculty of Biology, Medicine and Health, University of Manchester, Manchester, UK

The authors have addressed all my comments in my original review and I'm happy with this version.

I have read this submission. I believe that I have an appropriate level of expertise to confirm that it is of an acceptable scientific standard.

# Peer review: Reviewer response for version 2

**DOI:** 10.5256/f1000research.20430.r46713

**Published:** 2019-04-11

**Authors:** Patrick K. Kimes

**Affiliations:** 1 Department of Data Sciences, Dana-Farber Cancer Institute, Boston, MA, USA; 2 Department of Biostatistics, Harvard TH Chan School of Public Health, Boston, MA, USA

I appreciate the additional work done by the authors to address my comments from the earlier version of the manuscript. The new analyses and clarifications made to the text have fully addressed my previous concerns.

I have read this submission. I believe that I have an appropriate level of expertise to confirm that it is of an acceptable scientific standard.

# Peer review: Reviewer response for version 1

**DOI:** 10.5256/f1000research.18677.r42140

**Published:** 2019-01-18

**Authors:** Magnus Rattray

**Affiliations:** 1 Faculty of Biology, Medicine and Health, University of Manchester, Manchester, UK

Chabbert et al. provide an interesting study of the effect of gene model annotation on isoform abundance estimation from RNA-Seq data. They take a gene with 5 annotated alternative transcripts and look at expression over several tissues. They explore how well a transcript-based estimation method (Salmon) and differential transcript usage method (DRIMSeq) does when considering additional evidence from visualisation of read coverage and experimental validation of alternative transcripts by qPCR. They demonstrate that the inferred transcript abundance using the standard model is inconsistent with read coverage (e.g. an inferred highly expressed transcript has very low read density or junction read evidence for its only unique exon) and they show that better results are obtained when the model is modified by hand in a number of ways. They also show that through using their handcrafted gene model, consistency is improved when the dataset is changed to single-ended and shorter reads, which I found to be an interesting experiment. They also look at concordance across different RNA-Seq datasets measuring the same tissues and find the modification of UTRs in the gene model to improve concordance. 

 Overall I found this to be an interesting and useful contribution. Recent work (cited by the authors) has looked at metrics to identify discordance of the type observed here but nevertheless this careful study of one gene model is a useful contribution, especially give the experimental validation using qPCR. I think the data will be of great interest to methodology developers grappling with this problem although I guess long-read datasets may provide evidence for more genes in future studies than this kind of qPCR approach. 

 Major comments:

1.  As far as I can understand, in the end two kinds of change were made to the annotated gene model at the same time. A transcript Khk.RI was removed (this was tried first) and then both the UTRs were modified to be the same for all isoforms (first 3’ and then 5’) which means that new isoforms are added to the gene model and some were removed. The removal of a transcript strongly affecting results is arguably more problematic for transcript quantification methods because we would often expect the set of isoforms to contain unexpressed isoforms in a specific dataset. The other issue (an incomplete gene model) is already known to be problematic ^1^. However, I was not sure whether the UTR issue could potentially make the Khk.RI isoform issue worse. Therefore, I think it would be helpful to check whether Khk.RI inclusion remains problematic after making the UTR changes to the other isoforms. If adding an unexpressed isoform to the gene model can cause such an issue then this is a problem even when the gene model is correct. Also, there is a statement later on that “the removal of Khk.RI did further hinder reproducibility between both datasets” which seems to contradict the idea that removing this isoform is a good idea. I think this sentence is also a bit unclear and could do with some more explanation. 
2.  The modifications to the gene model in the end involves creating a new set of isoforms by combining existing exons and UTRs in a new configuration. Algorithms exist to do this in a data-driven way, e.g. the FlipFlop algorithm is designed to learn a set of isoforms from data by searching over all exon combinations using sparse inference on a splicing graph. Another example is StringTie and there may be later ones I'm not aware of. I think it would be useful to attempt to apply an algorithm of this type to this dataset to see how it would perform as this may provide a computational way to do something similar to what has been done manually in this example. Alternatively, these methods may fail and that would also be of interest. 

Minor comments:

1. Page 3 - “the recent development of alignment-free….has provided the possibility to quantify each individual transcript”. Methods that predate Salmon were already available for transcript quantification e.g. RSEM, eXpress, bitseq etc. I don't think we should confuse the speed-up introduced in Salmon and Kallisto with the transcript-level inference model introduced by RSEM and I think RSEM could have been used in this paper with similar conclusions. 
2. It would be good to know whether gene expression estimates based on the different gene models are performing differently, i.e. estimating gene expression by summing up isoform expression rather than counting. This has been discussed in the literature ^2^  and this issue could be explored in this dataset. 

Typos:

 Page 7 - “on estimate concordance” should be estimated.

I have read this submission. I believe that I have an appropriate level of expertise to confirm that it is of an acceptable scientific standard, however I have significant reservations, as outlined above.

## References

[1] SonesonCMatthesKLNowickaMLawCWRobinsonMD: Isoform prefiltering improves performance of count-based methods for analysis of differential transcript usage. *Genome Biol*.2016;17: 10.1186/s13059-015-0862-3 12 10.1186/s13059-015-0862-3 26813113PMC4729156

[2] SonesonCLoveMIRobinsonMD: Differential analyses for RNA-seq: transcript-level estimates improve gene-level inferences. *F1000Res*.2015;4: 10.12688/f1000research.7563.2 1521 10.12688/f1000research.7563.2 26925227PMC4712774

# Response

**Published:** 2019-03-18

**Authors:** Christophe Chabbert

**Affiliations:** ETH, Switzerland

We thank all the reviewers for their insightful comments and suggestions which have helped improve the quality of the manuscript. The main changes are summarized in the “Amendments from Version 1” section describing the new version of the manuscript. We will address the specific comments in this section.

**Major Point 1**

We have subjected the gene model annotation to two rounds of modifications to assess the impact of *Khk.RI* and UTRs on relative *Khk* isoform expression estimates. To assess the importance of the order in which these modifications happen, we have generated an additional annotation where *Khk.RI* is retained and all other *Khk* annotated transcripts have identical 5’ and 3’ UTRs. This annotation was used to estimate *Khk* isoform proportions in the Li et *al* (using the paired end and 50bp single-end data) and the Sollner et *al* datasets. We observed that implementing this new UTR model was not sufficient to remove the *Khk.RI* estimation bias in the paired-end data (updated Figure 3A). This bias was still particularly important in the bone marrow and spleen datasets where *Khk.RI* was estimated to account for more that 20% of the overall gene expression.

Comparison of the estimates between the paired-end dataset and the 50bp dataset derived from Li *et al* showed that this modification was however more beneficial than the removal of *Khk.RI* (Pearson correlation of 0.86 as opposed to 0.79 when *Khk.RI* is removed). Nevertheless, the best agreement between both libraries was still obtained after removal of *Khk.RI* and extension of the UTRs (Pearson correlation of 0.97).

Finally, comparison of the estimates of the Li et al and Sollner *et al* datasets revealed that the extension of the UTRs (Pearson correlation of 0.73) was more beneficial than *Khk.RI* removal (Pearson correlation of 0.46) but provided hardly any improvement over the naïve GENCODE annotations (Pearson correlation of 0.73). The complete set of modifications including *Khk.RI* removal and homogenization of the UTR was required to reach higher concordance between both datasets (Pearson correlation 0.91).

Overall, these observations suggest that both modifications are indeed needed to improve *Khk* transcript quantification results in the datasets considered in this study.

We have updated main Figure 3, main Figure 4 and the underlying data table 3 to report these findings. The result and discussion section of the article have also been modified accordingly:

“ *Additionally, we computed the relative Khk isoform usage using a new annotation with identical 5’ and 3’ end for all isoforms except Khk.RI which was retained as such in the gene model (Figure 3A). This modification was not sufficient to remove Khk.RI estimation biases, with the retained intron predicted to erroneously account for 20% of the overall gene expression in bone marrow or spleen. We therefore confirmed the impact and importance of both Khk.RI and UTRs annotations on isoform expression estimates for that gene.*”

“ *Both the removal of Khk.RI and UTR adjustments were necessary to reach this concordance between profiling methods*.”

Finally, we have modified the mentioned statement to emphasise the importance of performing both modifications on the concordance between estimates:

“ *While the removal of Khk.RI without an adjustment of the UTR did further hinder reproducibility between both datasets, we observed a much stronger consistency of proportion estimates when using the manually updated annotation*”

**Major Point 2**

Following this recommendation, we used StringTie as a complementary method to estimate transcript abundance across all tissues, with and without the reference annotation from GENCODE. Estimations output using the reference annotation still showed that the *Khk.RI* isoform still accounts for at least 15% of expressed isoform in 9 tissues. The results are presented in the Extended data, Figure 10A and the quantification results stored in the underlying data table 4. We also obtained StringTie quantifications without using a supporting genomic annotation, thereby allowing for the assembly of transcript by relying only on the current dataset. After merging the discovered transcript, we examined the resulting annotation for the *Khk* gene and compared it with the current GENCODE gene model (Extended data, Figure 10B). Quite interestingly, no corresponding *Khk.RI* isoform could be detected but *Khk.A*, *Khk.C* and *Khk.Skip* were all identified, albeit with UTR regions differing from the GENCODE annotation. No *KhkA.C* isoform could be detected while junction counts and coverage tracks indicate that it is expressed in low levels in certain tissues (liver or small intestine for example).

Taken together, these results suggest that, when relying on current annotations, StringTie is subject to similar biases as Salmon (in accordance with the observations made in Soneson et *al*) when it comes to the erroneous detection of the *Khk.RI* unexpressed transcript. When relying on transcript assembly, StringTie does provide different UTRs from the GENCODE annotation but fails to detect the *KhkAxC* isoform. As it is beyond the scope of this study to deliver a computational solution to what was achieved manually and at the single gene level, we have reported our observations but will not evaluate the feasibility of the scalability of an improved StringTie approach to tackle this problem.

We have modified the discussion section of the article as follows:

“ *To complement our main findings, we estimated transcripts abundance in the Li et al dataset using StringTie, as an example of tool relying on the construction of a splicing graph to quantify isoforms. When using the GENCODE annotation as a guide for quantification, we found that StringTie overestimated the Khk.RI expression in a fashion similar to Salmon. This is in concordance with the report from Soneson et al ^39^.* *In addition, we used StringTie without any supporting annotation to enable transcript assembly from the alignments. Inspection of the resulting newly assembled transcripts showed that Khk.RI could not be detected (Extended Data, Figure 10). Nevertheless, KhkA.C was also not identified in the dataset while junction counts clearly indicate that it could be detected in a handful of tissues, albeit with low expression levels. We therefore suggest that the issue reported here in the case of Salmon might be commonly found across quantification software, and it will be interesting to assess whether similar biases may arise with more tools. Results from Soneson et al. strongly indicate that this is the case, at least for a group of human genes ^39^ .* *”*

The method section has also been updated to document the parameters and settings used for the StringTie quantifications:

*“In order to quantify transcript using stringTie, alignments to the reference genome were first re-generated as described in the “RNA-seq data processing and alignment” section with the additional STAR flag “--outSAMstrandField intronMotif”. Quantifications were then performed with and without the GENCODE annotation used as a guide (-G option in stringTie). All other parameters were set to default values. Newly assembled transcripts were then merged using the transcript merge mode (--merge) with default parameters.* *”*

**Minor Point 1**

This is true and we have updated this section of the introduction to make sure this distinction is made clear. The section is now written as follows:

“ *In addition, the recent development of alignment-free transcript quantification methods has provided the possibility to efficiently and rapidly quantify each individual transcript ^15^^– 19 ^. Such approaches are indeed computationally much less demanding and faster than alignment-based methods ^15^ . Moreover, they have been shown to overcome the difficulty of handling multi-mapped reads, which can create biased results in count-based analysis ^21^*”

**Minor Point 2**

This is a good suggestion and following this recommendation and the major point 1 raised by Patrick Kimes, we have used *tximport* to obtain gene level estimates based on Salmon transcript quantifications from 3 of the modified annotations and the original GENCODE annotation. The results are now presented in the Extended Data, Figure 7. We found the gene level estimates to be consistent across all annotations and tissues and the patterns of expression across tissues to be conserved. In particular, *Khk* was found to be mostly expressed in liver, kidney and small intestine and that its expression was strongly tissue specific (adjusted p-value of 0 using the LRM from DESeq2 as described in the first version of the article). We have consequently added the following paragraph in the result section:

*“Finally, we evaluated whether the changes brought to the Khk transcript annotations affected the estimations of overall Khk expression levels. Gene level estimates obtained using quantifications based on the 3 modified annotations were strongly correlated (Pearson, r > 0.99) with estimates derived from the naïve GENCODE annotation (Extended data: Figure 7A). In addition, the high tissue specificity of Khk expression was observed in all cases, with expression patterns identical between annotations (Figure 1A, Extended data: Figure 7B).”*

# Peer review: Reviewer response for version 1

**DOI:** 10.5256/f1000research.18677.r42139

**Published:** 2019-01-16

**Authors:** Stephen N Floor, Luisa Mayumi Arake de Tacca

**Affiliations:** 1 Department of Cell and Tissue Biology (CTB), University of California, San Francisco (UCSF), San Francisco, CA, USA; 2 Comparative Biochemistry Program, University of California, Berkeley, Berkeley, CA, USA

Chabbert and colleagues did intricate work to measure how erroneous gene annotations without careful curation can lead to errors in gene expression analysis. This is a recurring problem in analysis of gene expression that has recently started to be addressed. In this work, they used a mouse gene (ketohexokinase) as a model to analyze publicly available data employing current alignment-free approaches to transcript quantification. The authors bring a real problem to the table and presents a good alternative as to how to proceed before analysis. Thanks for the deep study on how isoform annotations can affect gene expression quantification.

 Comments –

- In Figure 2, is it possible to show the same coverage tracks and assayed tissues (2B vs 2C)? What does the R1 isoform look like in the spleen? Do the authors have any theories about why the software is misusing the retained intron information during quantification?
- Why might correcting 3’ UTRs fix the problem? Would manually curating the 3’ UTR for each isoform make a difference? Same question for the 5’ UTR. A lot of times, the difference in isoforms is in transcription start sites, which have been observed to relate to downstream transcript processing events including splicing and poly-A site selection.
- Do the authors have any theories about how to generalize this approach for studies about many genes?
- How do the annotations for Khk change between databases? Would a unified annotation set using data from beyond Ensembl improve isoform quantification?

We have read this submission. We believe that we have an appropriate level of expertise to confirm that it is of an acceptable scientific standard, however we have significant reservations, as outlined above.

# Response

**Published:** 2019-03-18

**Authors:** Christophe Chabbert

**Affiliations:** ETH, Switzerland

We thank all the reviewers for their insightful comments and suggestions which have helped improve the quality of the manuscript. The main changes are summarized in the “Amendments from Version 1” section describing the new version of the manuscript. We will address the specific comments in this section.

**Point 1**:

The coverage tracks for all available tissues are shown either in Figure 2 or in the Extended Data, Figure 3 (accessible via this link - please make sure to expand the explorer tree on the left to make all files visible).

In this study we focused on identifying an example of gene model configuration that might be problematic. Although identifying which aspects of the quantification algorithms are causing this issue is definitely of interest, this question is beyond the scope of this study. Nevertheless, we invite the reviewers to consult the paper from Soneson et *al* as it provides additional insights into this aspect and explores other parameters, such as the choice of quantification method. We are hoping that the bioinformatics community will be able to identify the root cause of these biases.

**Point 2**:

This study focuses on providing an example of quantification bias and on highlighting some aspects of a gene model that have a strong impact on this bias. Identifying the algorithmic cause of the problem would be very interesting but is unfortunately beyond the scope of the work reported here. In the example of *Khk*, we have shown in this paper that the use of homogenous 3’ UTRs and 5’ UTRs across transcripts has helped overcome the quantification issue. Additional work will be required to determine whether that would be the case for all problematic genes and but this is also not part of the current study.

As pointed out, some reports have indeed shown that changes in 5’ or 3’ end are an important source of isoform diversity, which might complicate the interpretation of quantification results. As we mentioned in the abstract and in the discussion, it will be important to always thoroughly confirm such findings derived from transcript quantification studies. In addition, it is important to note that standard RNA-seq protocols are usually not suited to identify such changes in 5’ or 3’ end usage. Indeed, it is recommended to use protocols such as CAGE or 3’T-fill capture that can unambiguously identify transcription start or end. Therefore, such cases should be handled with an adequate experimental setting rather than standard RNA-seq strategies.

**Point 3**:

As previously commented in Points 1 and 2, our study did not aim at providing a generalized method that identifies problematic genes such as *Khk* or to provide a universal solution to the issue. The study from Soneson et *al* introduces the JCC index, which should be very helpful to identify problematic genes. In addition, in order to reduce the risk of making erroneous conclusions from large data analyses, we recommend confirming important findings using orthogonal methods (as stated in the abstract) such as count based approaches or well-targeted low throughput experiments such as qPCR for example.

**Point 4**:

In mouse, *Khk* annotations differ between Ensembl and RefSeq (please see the RefSeq annotation here, curated transcript NM_). When focusing on the curated transcripts, the most striking differences with the GENCODE annotation are the lack of *Khk.RI, Khk.Skip *and identical 5’ and 3’ UTRs. This would therefore be an equivalent situation to the one presented in this paper but without the *Khk.Skip* transcript available for quantification. This would therefore result in erroneous predictions as well. To comment on this point, we have added the following statement in the discussion section of the paper:

“ *It is also important to note that the curated RefSeq Khk gene model differs from the GENCODE as it is missing Khk.RI, Khk.Skip and the 3’ and 5’ UTRs of all curated transcripts are identical. While this would be a close configuration to the optimal annotation presented in this study, the absence of Khk.Skip in the gene model would result in erroneous quantifications as well.*”

While a consolidated annotation based on transcript model derived from large datasets might be an appealing idea, recent results from Soneson et *al* showed that in practice, this did not improve transcript estimate accuracy for problematic genes. We updated the discussion section of the paper in order to elaborate on this matter:

*“Improvement of current genomic annotations might ultimately offer an alternative as they will allow for the sole use of fast quantification algorithms. This might partially be achieved using transcript catalogues obtained from large scale studies such as CHESS even though Soneson et al reported very little to no improvement in their JCC scores using these new annotations.”*

# Peer review: Reviewer response for version 1

**DOI:** 10.5256/f1000research.18677.r42141

**Published:** 2019-01-08

**Authors:** Patrick K. Kimes

**Affiliations:** 1 Department of Data Sciences, Dana-Farber Cancer Institute, Boston, MA, USA; 2 Department of Biostatistics, Harvard TH Chan School of Public Health, Boston, MA, USA

Using the example of murine ketohexokinase (Khk), the authors present an analysis of the limitations of a current alignment-free approach (Salmon) to isoform-level quantification using RNA-seq data. In particular, using publicly available data sets, primarily from the Mouse BodyMap project ^1^, the authors show that transcript quantification using Salmon is highly sensitive to the specified reference transcriptome. These results are corroborated using qRT-PCR measurements of relative isoform abundance across several mouse tissues included in the study. 

 The authors apply two approaches to modify the commonly used GENCODE annotations for Khk to obtain improved estimates of isoform abundance using Salmon. First, the authors consider removing an annotated transcript, Khk.RI, with low support in unique exonic regions. Second, the authors modify the original annotations by trimming all 5' and 3' differences between isoforms. The authors show that with these two procedures, the resulting isoform estimates are more consistent across library protocols and across experiments - comparing against data from a separate study of mouse tissues ^2^. The advantage of removing weakly supported isoforms is in line with previous work which showed the benefits of prefiltering isoforms for false discovery rate control in studies of differential transcript usage ^3^.

 The conclusion is the need for "manual curation" of transcript annotations to improve quantification by current alignment-free approaches, and no general approaches are provided for improving all annotations. This is fine, as providing general guidelines (aside from careful review of isoform annotations) does not appear to be the purpose of this article.

 Overall, the paper provides an interesting and detailed analysis of a single gene that complements the growing literature on challenges in isoform-level quantification with RNA-seq. However, a few issues should be addressed before the paper and the analysis can be considered complete. These issues and a few other minor points are described below.

**Major Issues**

1. It appears that all gene-level analyses were carried out using gene count tables obtained using the QoRTs software, presumably based on the original ("naive") GENCODE transcript annotations. However, as one of the references cited by the authors points out ^4^, isoform-level quantification provides improved estimates of gene expression over simple count-based approaches. I am curious to see how the Salmon-based estimates (using tximport) of gene-level expression 1) compare with the QoRTs count tables, and 2) change with modifications to the transcript annotations. While the modified annotations appear to improve isoform-level estimates, it is also important to verify that they do not reduce the accuracy of gene-level estimates. This would further confirm the "reliability of gene expression", as described by the authors.
2. In the analysis of Salmon-based isoform quantification results, the authors claim that "inspection of the junction reads and coverage tracks revealed discrepancies between quantification estimates and expected results for several tissues." How were the expected results determined? From the text, this appears to be based on visual inspection of the coverage plots. However, per-base coverage can be tricky to visually interpret due to biases in RNA-seq data, e.g. sequence specific bias and fragment-level GC bias ^5^. Ideally, the definition of "expected results" should be made more concrete, e.g. using a metric such as the expected bias-corrected junction coverage as in Soneson et al., (2018) ^6^ or quantification of exon coverage as in Figure 1C. Regardless, the wording should be updated to more clearly state what constitutes "expected results" and how they were determined.
3. While the authors claim that "differences in 5' and 3' end annotations between all isoforms ... were not reflected on our coverage tracks," this does not appear to be true. In fact, there appears to be a clear difference in TSS coverage between tissues (Figure 3B). Notably, differences in 5' coverage appear to correspond to differences in coverage of the Khk.Skip splice junction (in agreement with GENCODE annotations). Additionally, clear TSS coverage differences were also observed and noted in the exon-level count analysis (Figure 1C), further supporting the presence and importance of TSS differences. The problem appears to be a mis-annotation of the 5' start site for the KhkC isoform (see Figure 1C where liver and kidney, two tissues with high KhkC preference according to RT-qPCR analysis, show significantly lower coverage in alternate start sites E01-E03). In light of these observations, the decision to completely trim all 5' and 3' differences seems rather extreme, and referring to the trimmed annotations as a "correction" of the gene models may not be accurate. Especially since such a trimming would result in the complete loss of the differential 5' behavior discovered earlier in the manuscript. This is particularly important as others have shown that start and termination site differences are the primary sources of isoform differences across human tissues ^7^. More justification and careful discussion of the limitations of this general trimming procedure are needed. Rather than a "correction", this appears to be a particularly aggressive modification that works in this setting because all isoforms differ by more than just the trimmed 5' and 3' regions (and not because "differences were not reflected on our coverage tracks", as stated in the text).
4. In modifying the GENCODE annotations, the authors use two steps. First, the removal of unexpressed transcript annotations, and second, the trimming of 5' and 3' differences. Figure 4D-F shows that simply removing the Khk.RI isoform from the annotations actually reduces the agreement between the two data sets, while additionally trimming 5' and 3' ends appears to greatly improve agreement. Have the authors considered how estimates change by applying the 5' and 3' trimming procedure without removing the Khk.RI transcript from the annotations? From the current analysis, it is unclear how much removing Khk.RI is improving the final result (with trimming), because these changes are not additive. 
5. While details are included for the Khk A/C-/- negative control mice, details on the non-control mouse tissue samples used for RT-PCR and qRT-PCR analyses are missing. How were these samples obtained and matched with the publicly available Mouse BodyMap RNA-seq data? This should be described in the Methods section.

  

**Minor Issues**

1. The authors claim that alignment-free transcript quantification methods have "provided the possibility to quantify each individual transcript", and that "this task is often impossible to complete using traditional count approaches." However, several methods for transcript quantification predate alignment-free methods, including some referenced later in the same section (Cufflinks, casper, FlipFlop), among others (RSEM, eXpress). It is also unclear what is meant by "often impossible." The wording in this section should be clarified.
2. It is unclear what "one common approach" is referencing at the beginning of the second paragraph describing differential gene expression (DGE) analysis. This is particularly jarring as the previous paragraph ends with noting the challenges of studying "complex events such as splicing or isoform usage switch". DGE analysis does not address these questions.
3. The authors claim that "DESeq2-tximport and sleuth [incorporate] estimates of inferential variances obtained during the quantification". While this is true of sleuth, I do not believe this is true of DESeq2-tximport. If it is, an appropriate reference should be added (none of the currently cited references describe this feature). Additionally, the reference linked to DESeq2-tximport (reference 22 in the article) ^8^ describes benchmarking several DGE methods to scRNA-seq data, and is a primary source for neither DESeq2 nor tximport. The more relevant reference would seem to be (reference 20 in the article) ^4^ which describes the tximport software package.
4. It is unclear what is being referred to by "these configurations", when claiming "no systematic evaluation of the impact of these configurations has been conducted."
5. The figure referenced in ".. hardly any detectable expression of the transcript (Figure 2B ...", should be "Figure 2C". It would also be helpful if the exon ID from the figure (E012) was also included in the text for easier reference.
6. Description and axes of Figure 3C,D should be corrected to reflect the fact that KhkA and KhkC estimates are actually (KhkA + KhkA.C) and (KhkC + KhkA.C) estimates, as described in the text.
7. In the Results section, "junctionSeq" should be stylized "JunctionSeq" for consistency.
8. Throughout, "Genecode" is probably meant to be "GENCODE" (or "Gencode").

I have read this submission. I believe that I have an appropriate level of expertise to confirm that it is of an acceptable scientific standard, however I have significant reservations, as outlined above.

## References

[1] LiBQingTZhuJWenZYuYFukumuraRZhengYGondoYShiL: A Comprehensive Mouse Transcriptomic BodyMap across 17 Tissues by RNA-seq. *Scientific Reports*.2017;7(1) : 10.1038/s41598-017-04520-z 10.1038/s41598-017-04520-z 28646208PMC5482823

[2] SöllnerJLeparcGHildebrandtTKleinHThomasLStupkaESimonE: An RNA-Seq atlas of gene expression in mouse and rat normal tissues. *Scientific Data*.2017;4: 10.1038/sdata.2017.185 10.1038/sdata.2017.185 PMC572631329231921

[3] SonesonCMatthesKLNowickaMLawCWRobinsonMD: Isoform prefiltering improves performance of count-based methods for analysis of differential transcript usage. *Genome Biol*.2016;17: 10.1186/s13059-015-0862-3 12 10.1186/s13059-015-0862-3 26813113PMC4729156

[4] SonesonCLoveMIRobinsonMD: Differential analyses for RNA-seq: transcript-level estimates improve gene-level inferences. *F1000Res*.2015;4: 10.12688/f1000research.7563.2 1521 10.12688/f1000research.7563.2 26925227PMC4712774

[5] LoveMIHogeneschJBIrizarryRA: Modeling of RNA-seq fragment sequence bias reduces systematic errors in transcript abundance estimation. *Nat Biotechnol*.2016;34(12) : 10.1038/nbt.3682 1287-1291 10.1038/nbt.3682 27669167PMC5143225

[6] SonesonCLoveMPatroRHussainSMalhotraDRobinsonM: A junction coverage compatibility score to quantify the reliability of transcript abundance estimates and annotation catalogs. *bioRxiv*.2018; 10.1101/378539 10.1101/378539 PMC633773930655364

[7] ReyesAHuberW: Alternative start and termination sites of transcription drive most transcript isoform differences across human tissues. *Nucleic Acids Res*.2018;46(2) : 10.1093/nar/gkx1165 582-592 10.1093/nar/gkx1165 29202200PMC5778607

[8] SonesonCRobinsonMD: Bias, robustness and scalability in single-cell differential expression analysis. *Nat Methods*.2018;15(4) : 10.1038/nmeth.4612 255-261 10.1038/nmeth.4612 29481549

[9] LiBDeweyCN: RSEM: accurate transcript quantification from RNA-Seq data with or without a reference genome. *BMC Bioinformatics*.2011;12: 10.1186/1471-2105-12-323 323 10.1186/1471-2105-12-323 21816040PMC3163565

[10] RobertsAPachterL: Streaming fragment assignment for real-time analysis of sequencing experiments. *Nat Methods*.2013;10(1) : 10.1038/nmeth.2251 71-3 10.1038/nmeth.2251 23160280PMC3880119

[11] GlausPHonkelaARattrayM: Identifying differentially expressed transcripts from RNA-seq data with biological variation. *Bioinformatics*.2012;28(13) : 10.1093/bioinformatics/bts260 1721-8 10.1093/bioinformatics/bts260 22563066PMC3381971

# Response

**Published:** 2019-03-17

**Authors:** Christophe Chabbert

**Affiliations:** ETH, Switzerland

We thank all the reviewers for their insightful comments and suggestions which have helped improve the quality of the manuscript. The main changes are summarized in the “Amendments from Version 1” section describing the new version of the manuscript. We will address the specific comments in this section.

**Major Point 1**

Thank you for making this suggestion. In order to address this point, we have used tximport to compute *Khk* expression levels from salmon estimates generated using the naive and all modified annotations (including the new one added to this version, see major comment 4). A comparison between the naïve annotation estimates and QoRTs counts is provided in the extended data, Figure 1B. A comparison between estimates from all tested annotations is now presented in the extended data, Figure 7 A and B. Examination of the tissue expression patterns of *Khk* (Panel A) and the correlation between gene level estimates clearly show a very good agreement between all annotations, thereby suggesting that the modifications have little impact on these estimates. We also verified that the very high tissue specificity of *Khk* in every case (adjusted p-value of 0 with DESeq2, following the similar analytical framework as described in the first version of the manuscript).

In order to clarify this point, we have added the following statement in the result section:

*“These results were all confirmed using the gene level estimates based on Salmon quantifications (Extended Data Figure 1A and B). In particular, patterns of Khk expression across tissues were highly concordant between count-based and Salmon estimates.”*

*“Finally, we evaluated whether the changes brought to the Khk transcript annotations affected the estimations of overall Khk expression levels. Gene level estimates obtained using quantifications based on the 3 modified annotations were strongly correlated (Pearson, r > 0.99) with estimates derived from the naïve GENCODE annotation (Extended data: Figure 7A). In addition, the high tissue specificity of Khk expression was observed in all cases, with identical expression patterns between annotations (Figure 1A, Extended data: Figure 7B).”*

**Major Point 2**

The expected results were determined using an inspection of the coverage tracks, the normalized exon coverage results obtained QoRTs and DEXSeq and previous reports from single gene studies. We have modified the wording in this section:

*“Despite this adjustment, inspection of the junction reads, coverage tracks, and normalised exon and junction counts derived from QoRTs (Figure 1C) revealed discrepancies between quantification estimates and results derived from alignment-based methods (Figure 3A and 3B). These quantification estimates also did not reflect the observations made by previous reports focusing on the characterisation of Khk expression patterns ^36^. An example of such discrepancy may be found examining the heart coverage tracks”*

**Major Point 3**

Thank you for pointing to this lack of clarity in the manuscript. It is indeed not the objective of this manuscript to suggest that a trimming or a standard homogenisation of the UTRs of all annotated transcripts of a gene will improve quantification results. Such an update of the annotation is possible because all *Khk* isoforms can be detected unambiguously just by considering the exclusion or inclusion levels of exon 3A and 3C (as partially stated in the discussion section). We have now made this clearer in the result section as well and explicitly mentioned this idiosyncrasy of the *Khk* gene model. We have also expanded the discussion section to elaborate on the limitations of this method for cases where UTRs are the main source of variability between isoforms of the same gene.

Finally, we would like to emphasize that the term correction is never used in this result section. In the title and abstract of this work, it refers primarily to the removal of the *Khk.RI* which is indeed a correction as this transcript expression levels remain below the threshold of detectability in the highly sensitive RT-qPCR assays.

The newly modified result section now reads as follows:

*“* *Since such quantifications are relying on the reference transcripts provided during the indexing step ^15^ , we reasoned that incorrect transcript models might be the cause of the observed discrepancies and therefore compared coverage tracks, normalized exon counts and isoform models. We identified annotated differences in 3’ end annotations between all isoforms which were not reflected on our coverage tracks (Figure 1C and Figure 3B). As Khk isoforms can be identified unambiguously based on the exclusion patterns of the exons 3A and 3C and regardless of differences in UTRs, we could investigate the impact of these UTR variations on transcript quantifications. We therefore manually updated Khk isoform annotations to provide an identical 3’ end to all isoforms (Underlying data: File 1 and 2) and re-estimated isoform proportions (Figure 3A, middle panel). Despite this adjustment, inspection of the proportion estimates still revealed erroneous estimations of isoform expression in particular in the case of liver where KhkA was detected in levels similar to KhkC. Further examination of the results revealed that, while some differences in 5’ end coverage in the dataset were concordant with the current gene annotation, they were not always reflected in the gene model (Figure 1C and Figure 3A, Heart, Lung and Spleen 5’ UTR coverage). Following a similar approach to the one described earlier for 3’ UTRs, we finally manually modified Khk isoforms to provide an identical 5’ and 3’ end to all listed isoforms (Underlying data: File 3).”*

The updated discussion section reads as follows:

“ *However, systematic harmonization of all UTRs across annotated transcripts might not be a general approach, especially in cases when such differences are reflecting tissue specific expression patterns*”

**Major Point 4**

To address this point, we have generated an additional annotation where *Khk.RI* is retained while all other *Khk* annotated transcripts have identical 5’ and 3’ UTRs. This annotation was used to estimate *Khk* isoform proportions in the *Li et al* (using the paired end and 50bp single-end data) and the Söllner *et al* datasets. We observed that implementing this new UTR model was not sufficient to remove the *Khk.RI* estimation bias in the paired-end data (updated Figure 3A). This bias was still particularly important in the bone marrow and spleen datasets where *Khk.RI* was estimated to account for more that 20% of the overall gene expression.

Comparison of the estimates between the paired-end dataset and the 50bp dataset derived from Li *et al* showed that this modification was however more beneficial than the removal of *Khk.RI* (Pearson correlation of 0.86 as opposed to 0.79 when *Khk.RI* is removed). Nevertheless, the best agreement between both libraries was still obtained after removal of *Khk.RI* and extension of the UTRs (Pearson correlation of 0.97).

Finally, comparison of the estimates of the Li *et al* and Söllner *et al* datasets revealed that extension of the UTRs (Pearson correlation of 0.73) was more beneficial than *Khk.RI* removal (Pearson correlation of 0.46) but provided hardly any improvement over the naïve GENCODE annotations (Pearson correlation of 0.73). The complete set of modifications including *Khk.RI* removal and homogenization of UTR was required to reach higher concordance between both datasets (Pearson correlation 0.91).

Overall, these observations suggest that both modifications are indeed needed to improve *Khk* transcript quantification results in the datasets considered in this study.

We have updated main Figure 3, main Figure 4 and the underlying data table 3 to report these findings. The result and discussion section of the article have also been modified accordingly:

“ *Additionally, we computed the relative Khk isoform usage using a new annotation with identical 5’ and 3’ end for all isoforms except Khk.RI which was retained as such in the gene model (Figure 3A). This modification was not sufficient to remove Khk.RI estimation biases, with the retained intron predicted to erroneously account for 20% of the overall gene expression in bone marrow or spleen. We therefore confirmed the impact and importance of both Khk.RI and UTRs annotations on isoform expression estimates for that gene.*”

“ *Both the removal of Khk.RI and UTR adjustments were necessary to reach this concordance between profiling methods*.”

**Major Point 5**

ll mice used in this study, C57BL/6J control and *KhkA/C ^-/-^*mice, were treated in a similar fashion and tissue were harvested on the same day. Additionally, RNA extraction, cDNA synthesis and gene expression analysis via RT-qPCR and semiquantitative RT-PCR were performed within the same experimental run. Differences in mRNA expression for *Khk* and *Khk* isoforms were evaluated via normalization to the internal reference gene *β-actin* for each sample. The wording in the methods section of the article has been modified accordingly:

*“C57BL/6J were obtained from The Jackson Laboratory while KhkA/C ^-/-^ mice, which are of C57BL/6 background and are lacking both ketohexokinase-A and ketohexokinase-C, were obtained from R. Johnson (University of Colorado) and used as negative control. All mice were housed in a pathogen-free facility at the ETH Phenomics Center (EPIC) under standard conditions (12 h light and 12 h dark cycle) with free access to food and water.”*

*“Total RNA from C57BL/6J and KhkA/C ^-/- ^mice was prepared from frozen tissues with RNeasy Mini Kit (QIAGEN, Hilden, Germany) and treated with DNase I to remove traces of DNA.”*

**Minor Point 1**

This sentence is indeed confusing and misleading and has therefore been removed from the introduction.

**Minor Point 2**

We have modified the start of the second paragraph to make the statement clearer:

“ *One common approach to analyse RNA-seq datasets consists in identifying significant changes in expression levels between two or more experimental conditions using gene-level counts*”

**Minor point 3**

Indeed, DESeq2-tximport cannot incorporate estimates of inferential variances. We have therefore updated the statement and corrected the reference, which should be the publication describing the tximport package.

**Minor point 4**

We have corrected the sentence to hopefully clarify its meaning:

“ *To our knowledge, no systematic evaluation of the impact of the presence of low coverage on such key regions has been conducted and detailed reports of such examples in real datasets are still missing*.”

**Minor point 5**

We have updated the figure reference in the main text and referenced the exon E012.

**Minor point 6, 7, 8**

Thank you for taking the time to highlight these discrepancies. We have corrected the wording in the Figure and the changed the “JunctionSeq” and “GENCODE” formatting in the main text.
